# Prenatal Origins of Temperament: Fetal Growth, Brain Structure, and Inhibitory Control in Adolescence

**DOI:** 10.1371/journal.pone.0096715

**Published:** 2014-05-06

**Authors:** Wolff Schlotz, Keith M. Godfrey, David I. Phillips

**Affiliations:** 1 Institute of Psychology, University of Regensburg, Regensburg, Germany; 2 MRC Lifecourse Epidemiology Unit, Southampton, United Kingdom; 3 Faculty of Social and Human Sciences, University of Southampton, Southampton, United Kingdom; 4 NIHR Southampton Biomedical Research Centre, University of Southampton and University Hospital Southampton NHS Foundation Trust, Southampton, United Kingdom; VU University Medical Center, Netherlands

## Abstract

**Objective:**

Individual differences in the temperamental dimension of effortful control are constitutionally based and have been associated with an adverse prenatal developmental environment, with structural brain alterations presenting a potential mechanism. We investigated this hypothesis for anatomically defined brain regions implicated in cognitive and inhibitory motor control.

**Methods:**

Twenty-seven 15–16 year old participants with low, medium, or high fetal growth were selected from a longitudinal birth cohort to maximize variation and represent the full normal spectrum of fetal growth. Outcome measures were parent ratings of attention and inhibitory control, thickness and surface area of the orbitofrontal cortex (lateral (LOFC) and medial (MOFC)) and right inferior frontal gyrus (rIFG), and volumetric measures of the striatum and amygdala.

**Results:**

Lower birth weight was associated with lower inhibitory control, smaller surface area of LOFC, MOFC and rIFG, lower caudate volume, and thicker MOFC. A mediation model found a significant indirect effect of birth weight on inhibitory control via caudate volume.

**Conclusions:**

Our findings support a neuroanatomical mechanism underlying potential long-term consequences of an adverse fetal developmental environment for behavioral inhibitory control in adolescence and have implications for understanding putative prenatal developmental origins of externalizing behavioral problems and self-control.

## Introduction

Effortful control (EC) is a temperamental dimension that describes individual differences in self-regulation, or the ability to exert behavioral control, including attentional focusing and inhibitory control [1]. Children and adolescents high in EC are able to inhibit a dominant response or activate a subdominant response, to maintain a sustained focus of attention, and to plan behavior. Individual differences in EC are likely to affect individual adjustment, success, health and well-being later in life. For example, low EC has been shown to be a risk factor for both internalizing and externalizing problems [2], [3], [4], [5], [6], might have evocative effects on mother's teaching strategies [7], and influence academic development [8]. Recently, a related but broader construct of self-control in childhood, including lack of control, persistence and attention as well as impulsivity, has been shown to predict adult physical health, substance dependence, personal finances and criminal offending [9]. Because of such broad and substantial potential consequences, investigating the origins of individual differences in EC is of high relevance.

Differences in EC are thought to be constitutionally based, which implies a biological basis. It has been suggested that genetic factors as well as maturation and experience are the primary factors affecting individual differences in EC [1]. Besides these factors, individual differences in behavior and mental health have been associated with an adverse prenatal developmental environment [10], [11]. Birth weight across the full normal range is an indicator of prenatal adversity and has been associated with EC and executive functions in children [12], [13], [14]. In addition, a number of studies demonstrated associations of birth weight with symptoms of attention-deficit/hyperactivity disorder (ADHD) [12], [15], [16], [17], [18], [19], [20]. Because EC and ADHD symptoms are conceptually and empirically related [3], [21], we have suggested that EC might mediate the association between prenatal adversity and ADHD symptoms [12].

However, the mechanisms underlying the association between birth weight and EC are unknown. A number of studies recently demonstrated associations between birth weight and global and regional brain morphology across the full normal range of birth weight in broad age samples including children, adolescents and adults [22], [23], [24]. Structural changes in brain development might be associated with individual differences in temperamental traits such as EC later in life [12], [25]. In this study, we explore if specific alterations in brain structure might present a mechanism for putative fetal origins of EC in adolescence.

It has been suggested that EC is based on the executive attention network, which is involved in resolving conflict between other brain networks and is thought to underlie cognitive and emotional self-regulation [26]. Functional neuroimaging of activation during tasks that require cognitive control or response inhibition suggested that the executive attention network consists of the anterior cingulate cortex (ACC) and prefrontal, particularly orbitofrontal, cortical areas [26], [27]. Consistently, effortful control has been shown to be associated with left orbitofrontal cortex (OFC) volume in adolescents [25], and structural deficits in ADHD have repeatedly been observed in OFC and anterior cingulate areas [28].

However, as mentioned above, EC is a heterogeneous construct including lower order facets, particularly attention and inhibitory control. Akin to this heterogeneity it has been suggested that executive attention functions, reflected in anterior cingulate activation, should be distinguished from executive motor control [29], or, similarly, cognitive control from control over overt behavior, e.g. motor inhibition and impulse control [30]. Inhibitory motor control has been shown to implicate prefrontal cortical areas and fronto-striatal circuits, with the right inferior frontal gyrus (rIFG) and striatum, particularly caudate, thought to be important areas for effective response inhibition [31], [32], [33], [34], [35]. Support for the relevance of a fronto-striatal pathway in inhibitory motor control comes from observations of activation in the lateral and medial frontal cortex, putamen and caudate during response inhibition tasks [36], [37], [38], [39], [40], [41]; deficits in volumes of prefrontal cortical and basal ganglia structures in ADHD patients [28], [32], [42], [43]; and correlations of frontal gray matter and caudate volume with physician and parent ratings of ADHD symptoms [44]. In addition, higher ratings on nonplanning, motor and cognitive impulsivity were associated with higher bilateral caudate activity during an inhibitory control task [39] and lower OFC volume [45], [46], [47]. In support of the notion that prenatal factors might affect the development of this pathway, a recent study has demonstrated shape contraction and smaller volume of the caudate bilaterally in boys with relatively low birth weight born at a short gestational age [48], and birth weight was shown to be correlated with caudate volume in another study [22].

In addition, a number of studies suggested a neural basis for impulse control based on a circuit including ventromedial prefrontal cortex (VMPFC), anterior cingulate cortex (ACC) and amygdala [39], [49]. As impulsivity is a construct closely related to (low) inhibitory control, this circuit might also be implicated in long-term effects of prenatal adversity on inhibitory control, and recent data has reported a link between prenatal maternal depression and lower fractional anisotropy and axial diffusivity in the right amygdala of infants at birth (but no association with amygdala volume) [50], as well as associations between maternal cortisol levels in early gestation and child left amygdala volume at 7 years of age [51].

Based on these findings we hypothesized that an adverse prenatal developmental environment might lead to deficits in attention and inhibitory control in adolescence, and that such associations would be based on effects of prenatal adversity on brain development resulting in specific structural changes. The aim of our study was to investigate in a longitudinal birth cohort (1) whether birth weight across the normal spectrum (an indicator of fetal adversity) is associated with attention and inhibitory control in adolescence, and (2) if structural characteristics of OFC, rIFG, ACC, striatum and amygdala statistically mediate such associations.

## Materials and Methods

### Study design and participants

This is a follow-up study of children of mothers recruited for a longitudinal birth cohort study at 17 or less weeks of gestation at the Princess Anne Maternity Hospital in Southampton, UK [52]. A subgroup of 139 singleton children was recruited for a study when they were 7–9 years old [53], [54]. For the follow-up study reported here, families of this subsample were contacted again when the children were approximately 14–15 years old. The local NHS National Research Ethics Service Committee Oxfordshire REC B approved the study and both parent and children gave written informed consent. Participants received a reimbursement of £140 to cover travel costs and their time invested.

Data on birth weight and gestational age were used to generate groups of participants to be recruited for the MRI study with the aim of having the full normal spectrum of fetal growth represented by the sample. In a first step, five participants born before the 37th week of gestation were excluded to prevent any confounding effect of preterm birth. The remaining 134 participants were then allocated to five groups based on sex-specific fetal growth (FG; birth weight adjusted for gestational age) by regressing birth weight on gestational age and using quintiles of the residuals for group allocation separately for males and females. This resulted in equally sized groups of low FG (n = 27), low-medium FG (n = 27), medium FG (n = 27), medium-high FG (n = 27) and high FG (n = 26). To represent the full normal spectrum of FG and increase power of the hypothesis tests only those 80 participants in the low, medium and high FG groups were included in the study. Of those, addresses of 27 were not traceable; the remaining participants were contacted by telephone and screened for exclusion criteria. Twenty-four participants were excluded because they wore dental braces (n = 6), did not want to participate (n = 12), reported symptoms of claustrophobia (n = 2), had metal implants (n = 2) or suffered from epilepsy (n = 1), and one participant was surplus to requirements. Thus, 29 adolescents were recruited into the MRI study. After MRI scanning, one participant was excluded due to poor image quality, and one participant had a very small total brain volume at 2.5 SD below the sample mean and therefore was excluded from all brain analyses. Thus, the resulting total sample size was n = 27 (n_lowFG_ = 8; n_mediumFG_ = 12; n_highFG_ = 7). The 27 participants included in the study did not differ from the 53 excluded in terms of age, sex, birth weight, and gestational age (all *p*s>.24).

### Measurements

#### Data collected at birth

At birth, the infant's weight was measured using digital scales; the infant's gestational age at birth was calculated from the date of the last menstrual period, confirmed by ultrasound [52].

#### Temperamental factors

Inhibitory control and attention were measured by parent reports on two subscales of the Early Adolescent Temperament Questionnaire – Revised (EATQ-R) [55]. The scale Attention measures the capacity to focus and shift attention when desired (6 items; Cronbach's alpha  = .81). Item examples are “Finds it easy to really concentrate on a problem” and “When interrupted or distracted, forgets what s/he was about to say” (reverse scored). The scale Inhibitory Control measures the capacity to plan and to suppress inappropriate responses (5 items; Cronbach's alpha  = .58). Item examples are “Has a hard time waiting his/her turn to speak when excited” (reverse scored), and “Is usually able to stick with his/her plans and goals”. The scores on the two scales in this sample correlated significantly (r = .50, p = .006).

### MR imaging

Imaging data were acquired at the Oxford Centre for Clinical Magnetic Resonance Research, John Radcliffe Hospital, Oxford, UK, on a 3.0 Tesla TIM Trio scanner (Siemens, Erlangen, Germany). T1-weighted structural images for the structural analysis were acquired using a 3D magnetization prepared rapid gradient echo (MPRAGE) sequence. The voxel resolution was 1×1×1 mm^3^ with an acquisition matrix of 174×192×192 and the following parameters: TR = 2040 ms; TE = 4.7 ms; inversion time (TI)  = 900 ms; flip angle  = 8°. Acquisition duration for this sequence was 5 min and 56 s. The sequence was acquired twice during the same session for each adolescent.

### Image analysis

Cortical reconstruction and volumetric segmentation was performed with the FreeSurfer v5.1.0 imaging analysis suite (https://surfer.nmr.mgh.harvard.edu). This process included motion correction and averaging of the two T1-weighted images acquired for each subject, removal of non-brain tissue, and segmentation of white matter and subcortical grey matter volumetric structures [56]. Cortical thickness and surface area were obtained by reconstructing the grey/white matter boundary and the pial surface of the cortex [56]. Cortical thickness was calculated as the closest distance from the grey/white matter boundary to the pial surface at each surface location. Average cerebral cortical thickness and area for regions of interest (ROIs) were based on automated parcellation of the cortex into units based on gyral and sulcal structure using the Desikan-Killiany atlas [57]. Total brain volume was calculated by the volume of all brain labels [56].

**Figure 1:**
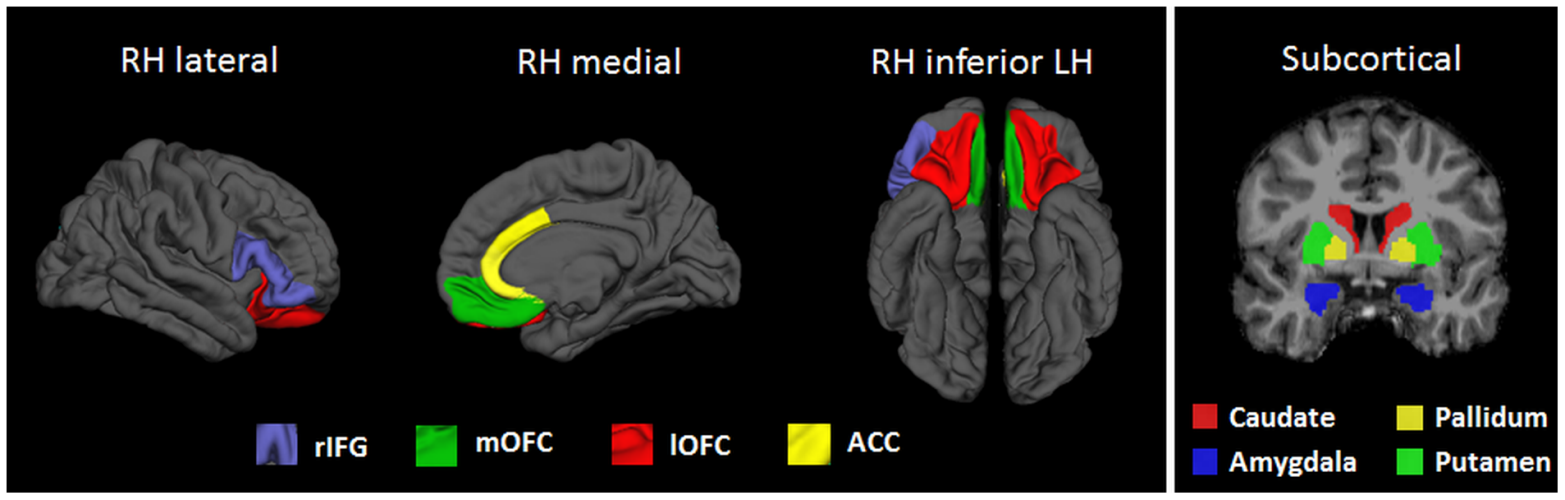
Cortical and subcortical anatomical target regions of interest (ROIs) for this study. LH: Left hemisphere; RH: Right hemisphere; rIFG: Right inferior frontal gyrus; LOFC: lateral orbitofrontal cortex; MOFC: medial orbitofrontal cortex; ACC: Anterior cingulate cortex.

Anatomical cortical ROIs were defined as the medial orbitofrontal cortex (MOFC), lateral orbitofrontal cortex (LOFC), ACC (comprising rostral and caudal anterior cingulate) and rIFG (comprising pars opercularis, pars triangularis and pars orbitalis). Exact anatomical boundaries and reliability of the parcellation are described elsewhere [57]. Average cortical thickness (CT) and surface area (SA) of these ROIs were used for hypothesis testing. Volumetric subcortical segmentation was based on validated automated procedures [56]; subcortical ROIs comprised the striatal areas of caudate, putamen and pallidum, and the amygdala. Figure 1 shows the ROIs for this study. Apart from rIGF, we used the total (left + right) CT, SA or volume of the structures, as we had no *a priori* hypotheses on hemisphere specificity. In addition, results of an exploratory whole-brain analysis are shown in the supporting information (for results of surface area analysis see Figure S1 in File S1; for results of cortical thickness analysis see Figures S2 and S3, both in File S1).

### Statistical analysis

The main analysis focused on a potential mediating effect of structural brain characteristics for associations between fetal adversity and behavioral measures of attention and inhibitory control. Indicators of average CT, SA and volume were exported from FreeSurfer into Stata v12.1 (StataCorp, College Station, Texas). We used birth weight as a continuous predictor of brain structure and behaviour in hierarchical least squares multiple linear regression models. Potential mediation effects were tested by estimating path models including indirect effects using Mplus v6.11 (Muthén & Muthén, Los Angeles, California). Tests were conducted in the following order. First, associations between birth weight and behavioral measures (attention, behavioral inhibition) were tested. Second, associations between fetal adversity and brain structure in adolescence were tested by regressing structural brain characteristics of the ROIs defined above on birth weight. Third, behavioral measures were regressed on those structural brain indicators that showed significant associations with birth weight in the step before. All three association tests should reveal statistically significant associations for a mediating effect to be plausible. Therefore, mediation models were tested only for those behavioral measures and brain structures that fulfilled these criteria. To control for potential sex differences in birth weight and brain structure, all analyses were adjusted for sex. Brain analyses were additionally adjusted for intracranial volume (ICV) by including ICV as a covariate into the regression. To explore potential prenatal and postnatal confounding variables, we also included maternal smoking and drinking alcohol during pregnancy, parity and social class as covariates in additional models. All hypotheses were tested using a significance level of p<.05.

## Results

Table 1 shows characteristics of the study participants. Gender proportions were similar in the three groups (Fisher's exact test, p = .90), and mean age did not significantly differ between the groups (F = 0.22, p = .80). As intended by design, groups had a similar mean gestational age (F = 0.32, p = .73), whereas mean birth weight differed between groups (Low v Medium FG: t = 2.67, p = .016; Low v High FG: t = 5.37, p<.001; Medium v High FG: t = 4.79, p<.001). Thus, the total sample represents fetal growth adjusted for gestational age across the full normal spectrum of birth weight.

**Table 1:** Characteristics of the sample as represented by three groups of fetal growth.

	Low FG group (n = 8)	Medium FG group (n = 12)	High FG group (n = 7)
Age (years)^a^	16.0 (0.36)	15.9 (0.41)	16.0 (0.34)
Age range (years)	15.4–16–6	15.1–16.4	15.5–16.4
Female^b^	3 (37%)	6 (50%)	4 (50%)
Birth weight (kg)^a^	3.2 (0.49)	3.5 (0.28)	4.1 (0.26)
Birth weight range (kg)	2.4–3.6	3.1–4.2	3.7–4.5
Gestational age (weeks)^a^	40.3 (1.56)	39.8 (1.36)	40.1 (1.56)
Gestational age range (weeks)	37.9–42.3	38.1–43.3	38.1–41.7

Note that the only statistically significant differences between groups was in birth weight (see text for details).

a M (SD);

b n (%); FG: Fetal growth.

### Birth weight and behavior

Birth weight significantly predicted inhibitory control (β = 0.41, p = .043, ΔR^2^ = .15), but not attention (β = 0.06, p = .76). Thus, consistent with our hypothesis, adolescents born at higher birth weight showed more behavior indicative of higher inhibitory control as reported by their parents, with birth weight explaining 15% of the variance in inhibitory control. In contrast, attention was not significantly related to fetal growth. Adjusting the models for maternal smoking, drinking alcohol, parity and social class did not change the results (supporting information, Table S1 in File S1).

### Birth weight and brain structure

Birth weight was positively associated with total brain volume (β = 0.33, p = .001, ΔR^2^ = .10), total gray matter (β = 0.26, p = .023, ΔR^2^ = .06) and white matter volume (β = 0.34, p = .022, ΔR^2^ = .10).

**Figure 2:**
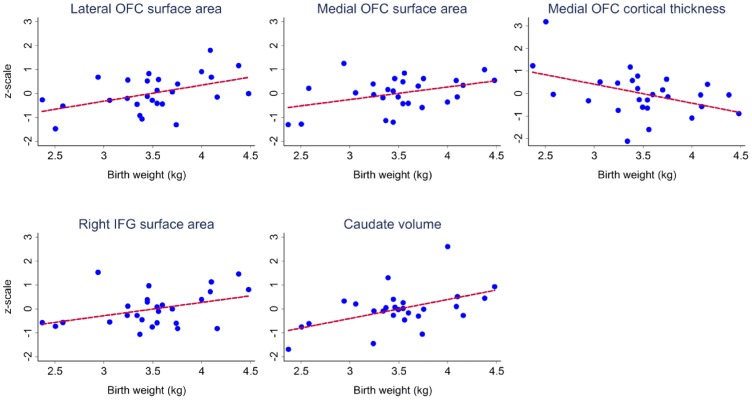
Scatter plots showing statistically significant associations between birth weight and neuroanatomical variables residualized for sex and intracranial volume.

Table 2 shows the results of regressions of cortical ROIs on birth weight. Whereas adolescents born at a higher birth weight showed larger SA in rIFG and both medial and lateral OFC, no significant associations were observed for ACC. Birth weight was also significantly negatively related to thickness of the medial OFC, explaining between 10 and 22% of variance in cortex morphology. Figure 2 illustrates the statistically significant associations found.

**Table 2:** Associations of birth weight with bilateral structural measures of cortical regions of interest.

	Surface area			Cortical thickness		
	β	p	ΔR^2^	β	p	ΔR^2^
LOFC	**0.45**	**.010**	**.17**	−0.24	.24	.05
MOFC	**0.35**	**.029**	**.11**	**−0.51**	**.016**	**.22**
rIFG	**0.35**	**.035**	**.10**	−0.02	.93	.00
ACC	0.24	.20	.05	−0.06	.79	.00

*Note*. Adjusted for sex and intracranial volume. LOFC: lateral orbitofrontal cortex; MOFC: medial orbitofrontal cortex; rIFG: Right inferior frontal gyrus; ACC: Anterior cingulate cortex.

Results of regression analysis testing associations of birth weight with volumes of subcortical ROIs are shown in Table 3. Caudate volume showed a significant positive association, with birth weight explaining 21% of the variance in caudate volume. This effect is illustrated Figure 2. In contrast, volumes of the other investigated subcortical structures were not significantly related to birth weight. Adjusting the models for maternal smoking, drinking alcohol, parity and social class did not change the results (supplemental material, Table S1 in File S1).

**Table 3:** Associations of birth weight with volume of subcortical gray matter regions of interest.

	β	p	ΔR^2^
Amygdala	0.03	.90	.00
Caudate	**0.50**	**.005**	**.21**
Putamen	−0.01	.95	.00
Pallidum	0.11	.55	.01

*Note*. Adjusted for sex and intracranial volume.

### Inhibitory control, birth weight and brain structure

Results so far suggest SA of rIFG, LOFC and MOFC, CT of MOFC, and caudate volume as potential mediators of the relationship between birth weight and inhibitory control. To establish a potential mediational path, we next tested for associations between these structural indices and inhibitory control scores. Regression models showed no significant relationship for any of the cortical structures, all explaining almost no variance in inhibitory control (rIFG SA: β = 0.06, p = .82, ΔR^2^ = .00; LOFC SA: β = 0.09, p = .71, ΔR^2^ = .01; MOFC SA: β = 0.05, p = .85, ΔR^2^ = .00; MOFC CT: β = −0.03, p = .89, ΔR^2^ = .00). In contrast, caudate volume was significantly and positively associated with inhibitory control, explaining 20% of variance (β = 0.53, p = .015, ΔR^2^ = .20), thus qualifying as a potential mediator of the relationship between birth weight and inhibitory control.

**Figure 3:**
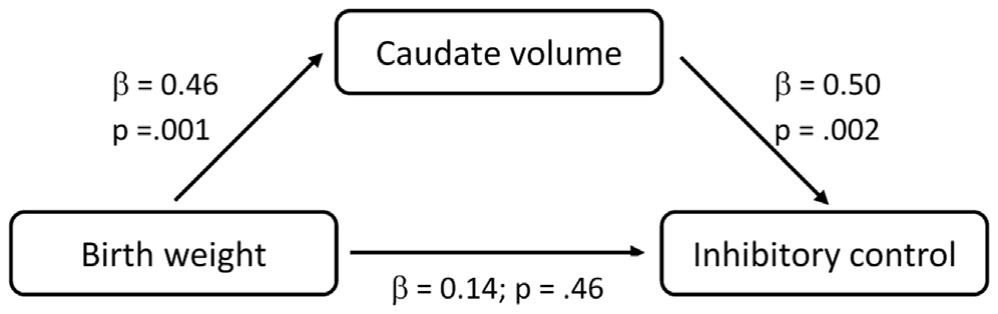
Mediation analysis for the effect of birth weight on inhibitory control via caudate volume. Model adjusted for sex and intracranial volume. Coefficients shown are standardized regression coefficients. The total indirect effect of birth weight on inhibitory control was statistically significant at p = .025. Omitting caudate volume from the analysis, birth weight was associated with inhibitory control, β = 0.41, p = .043.

A formal mediation test using path analysis tested the indirect effect of birth weight on inhibitory control via caudate volume (Figure 3). Confirming results presented above, paths from birth weight to caudate and from caudate to inhibitory control were highly significant, and the initially significant association between birth weight and inhibitory control became non-significant when caudate volume was entered in the model. The model confirmed that the indirect pathway effect from birth weight to inhibitory control via caudate volume was significant (indirect β = 0.29; p = .025).

## Discussion

The results of our study suggest that an adverse fetal developmental environment, indicated by fetal growth across the full normal spectrum, may affect inhibitory control in adolescence, and that this effect may be mediated through subtle changes in subcortical brain structures involved in executive motor control.

Of the two facets of effortful control studied here, only inhibitory control and not attention was associated with birth weight. Whether this is due to methodological factors (e.g. limited statistical power; parent report rating scales as opposed to self-report ratings or cognitive tests; differences in measurement reliability), or reflects specificity in long-term consequences of fetal adversity in adolescence is not clear. For inhibitory control we were able to detect a significant indirect effect of birth weight via caudate volume, suggesting that fetal adversity might be linked to behavior in adolescence via alterations in brain development.

We found that birth weight was positively associated with total brain, gray and white matter volume, as well as cortical surface area in both LOFC and MOFC, and in rIFG. With regards to subcortical structures, birth weight was positively associated with caudate volume, but not with other striatal areas or the amygdala. These findings are largely consistent with recent studies in larger samples that found birth weight effects for surface areas similar to our frontal cortical ROIs [22], [23], [24] as well as caudate volume [22], [48]. Associations in our study reflect relatively large effects with 10–22% of variance in brain structure explained by birth weight. In comparison, Walhovd and colleagues [22] reported effects that explained 3% of variance in total brain volume and 4% in caudate volume. A number of study design factors are possible reasons for the larger effects found in our study. First, we residualized birth weight for gestational age and our results therefore more accurately reflect effects of fetal growth. Second, we selected participants from three groups representing the full spectrum of fetal growth; including the extreme groups might have increased statistical power in our study. Finally, we used data from a longitudinal birth cohort with birth weight recordings done at birth by nurses using digital scales, as opposed to birth weights being recalled by parents or participants at the time of the neuroimaging session, thus reducing unsystematic variance due to recall bias. Nevertheless, our study still had limited statistical power due to the relatively small sample, which might explain some of our negative findings.

In contrast to cortical surface area, a significant association with cortical thickness was found only for the MOFC. Adolescents with lower fetal growth had a thicker MOFC averaged across hemispheres. This finding is consistent with earlier studies of samples covering childhood to young adult age groups showing that cortical thickness was not or only very weakly associated with indicators of fetal growth in both monozygotic and dizygotic twins [23], in a larger sample of adolescents [22], and in both schizophrenia patients and healthy controls [24]. In children and adolescents born small-for-gestational age, thickening of frontal cortical areas was found, although the effects where rather small [58], [59]. As brain development is characterized by progressive cortical thinning during adolescence [60] these results might indicate a maturational delay of orbitofrontal cortex in adolescents with low fetal growth. Alternatively, the weak associations found here might be due to opposing effects of fetal growth and overall brain size on cortical thickness. However, we also found birth weight to be more strongly, and positively, associated with white matter volume than gray matter volume. Although speculative, together with our cortical thickness finding this could indicate a less extensive architecture of white matter tracts in adolescents with low fetal growth, suggesting that future studies examining connectivity of gray and white matter areas using Diffusion Tensor Imaging might reveal important additional information on potential effects of fetal adversity on brain structure in adolescence.

It has been suggested that the striatum is particularly vulnerable to perinatal hypoxic-ischemic events [61] and intraventricular hemorrhage [33], leading to reduced cognitive and behavioral control in affected individuals. In addition, experimental animal studies have shown that an adverse intrauterine environment due to hypoxic-ischemic injury and inflammatory insults can lead to neuronal death, white matter damage, and reduced brain growth [62], [63]. In guinea pigs, reduced uteroplacental blood flow has been shown to lead to reduced brain weight and reduced basal ganglia volume lasting into adolescence [64]. Therefore, long-term consequences of fetal adversity are possible and the behavioral consequences suggested by our results are plausible. However, what remains unclear is to what extent our findings represent maturational delay or impairment of normal brain development. Also, our study does not allow any causal conclusions, as alternative explanations cannot be ruled out. It is well known that genetic and postnatal environmental factors contribute to brain development [65], [66]. As effects of birth weight on brain structure where demonstrated within monozygotic twin pairs [23], genetic effects seem to be an unlikely alternative explanation. Including maternal smoking and drinking alcohol, parity and social class in our models did not change any of the associations observed. However, continuation of adversity in postnatal life and residual confounding variables could not be controlled in more detail in our study.

The finding of a positive association between caudate volume and inhibitory control in daily life is consistent with neuroscience models that postulate a major involvement of striatal areas in inhibitory motor control [33], [34], [41], [67], [68] and findings of reduced caudate volume in ADHD [69]. However, contrary to our expectations, none of the other brain structures investigated were associated with inhibitory control. This could be due to limited reliability of the behavioral scale, limited statistical power, or it might indicate structural differences that do not translate into function. Using fMRI with an inhibitory control task could provide some clarification of this question, which might be highly relevant as the neural mechanisms of stopping have an effect on the broader construct of self-control [67], which itself is associated with behavioral problems, adjustment, and health and wealth later in life [2], [9].

The main limitations of our study were the relatively low internal consistency of the Inhibitory Control scale and limited statistical power due to a relatively small sample. The internal consistency of a scale is dependent on the number of items and the observed scale score variance. For a short scale of five items and a relatively homogenous and small sample the internal consistency observed in this study can be considered acceptable for a group study. Nevertheless, both limitations mean that some relevant associations might have been missed.

In summary, this study demonstrated that an adverse fetal developmental environment might lead to reduced inhibitory control in adolescence, and that reduced caudate volume might mediate this association. As inhibitory control is an integral part of self-control, identifying such pathways originating in prenatal life might in the future help to improve well-being through targeted early preventative action such as educational or nutritional intervention [70], [71], [72]. However, the exact mechanisms by which prenatal factors might affect behavior and well-being later in life first need to be defined more clearly. Concerning brain development, future studies need to investigate additional effects of fetal adversity such as alterations of neural connectivity and differential functional activation of potentially relevant areas.

## Supporting Information

File S1 **Combined supporting information, containing results of vertex-wise analyses (Figures S1, S2, and S3) and results of adjustment for potentially confounding covariates (Table S1).**
(DOCX)
