# Supplementary material for: Prenatal Origins of Temperament: Fetal Growth, Brain Structure, and Inhibitory Control in Adolescence
Source: PLoS One. 2014 May 6;9(5):e96715. doi: 10.1371/journal.pone.0096715 (PMC4011887; doi:10.1371/journal.pone.0096715)
Supplement: File S1 — Combined supporting information, containing results of vertex-wise analyses (Figures S1, S2, and S3) and results of adjustment for potentially confounding covariates (Table S1). (DOCX) [file pone.0096715.s001.docx]

**S1: Combined supporting information to:**

**Schlotz, W., Godfrey, K., & Phillips, D.I.W.: Prenatal origins of temperament: Fetal growth, brain structure, and inhibitory control in adolescence.**

***Results of vertex-wise analyses***

In the following, we present results of FreeSurfer vertex-wise whole-brain analyses of cortical surface area and thickness in our sample of n = 27 participants (see Methods section for sample details). All analyses were initially thresholded at an uncorrected error rate of p < .01. This initial analysis was followed up by a correction for multiple comparisons, i.e. setting the false discovery rate (FDR) at FDR at 5%.

Figure S1 shows associations of birth weight with cortical surface area, adjusted for sex, thresholded at an uncorrected p < .01, smoothed by full width half maximum (FWHM) at 15 mm. There were a few weak negative associations in temporal, frontal and occipital areas. However, none of these associations remained significant when controlling for multiple comparisons.


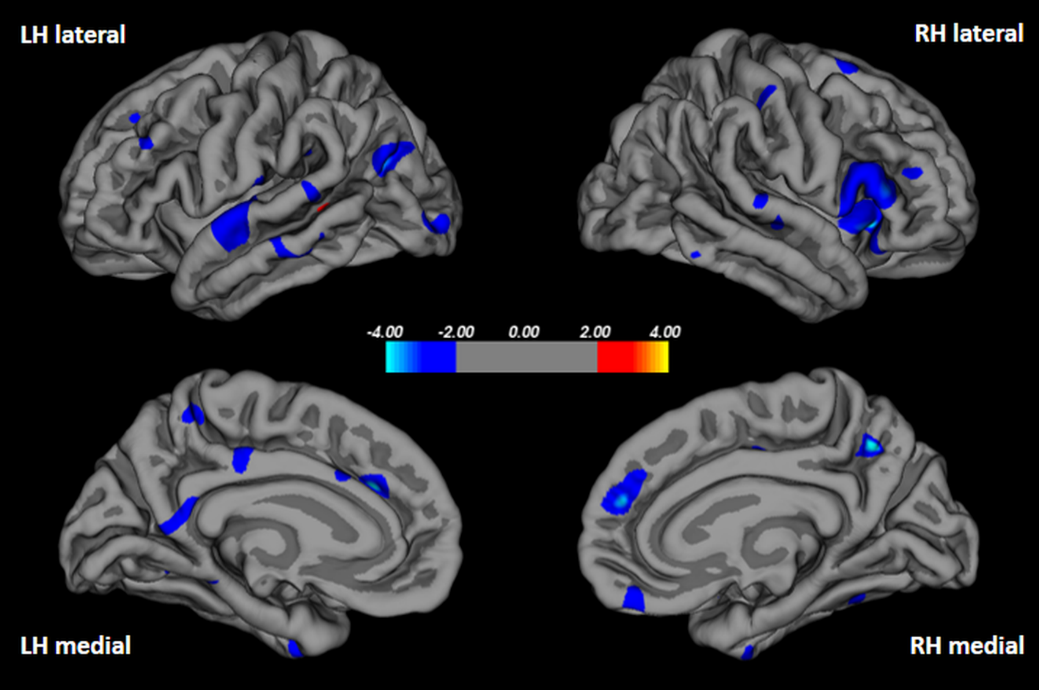
**Figure S1.** Results of vertex-wise analysis of birth weight with cortical surface area (adjusted for sex) at p < .01, uncorrected, FWHM = 15 mm. Significance shown is a –log(10)p value.

Figure S2 shows associations of birth weight with cortical thickness, adjusted for sex at p < .01 (uncorrected). There were a few weak negative associations in left occipital areas, and one relatively strong negative association of right medialorbitofrontal cortical thickness with birth weight, which remained significant when controlling for multiple comparisons (see Figure S3).


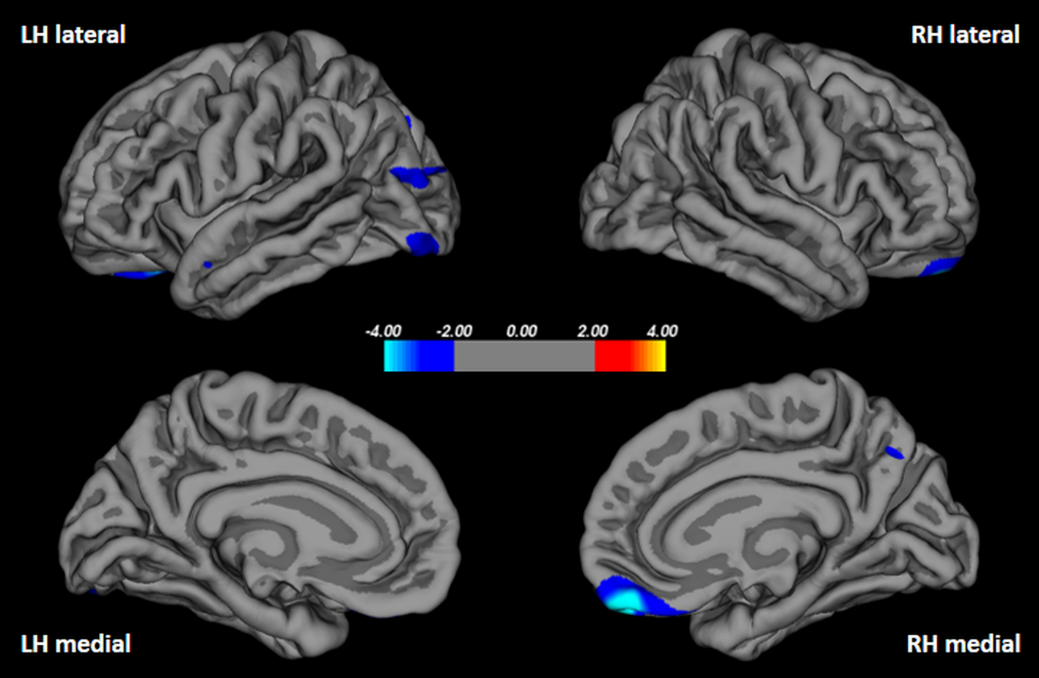
**Figure S2.** Results of vertex-wise analysis of birth weight with cortical thickness (adjusted for sex) at p < .01, uncorrected. Significance shown is a –log(10)p value.


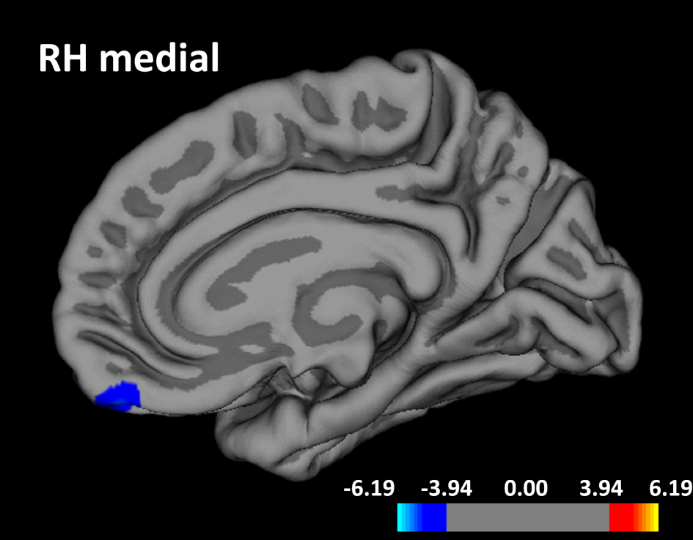
**Figure S3**. Right hemisphere, medial view of results of vertex-wise analysis of birth weight with cortical thickness (adjusted for sex), FDR threshold = .05. Significance shown is a –log(10)p value.

***Results of adjustment for potentially confounding covariates***

In the following, we present results of regression analyses with additional adjustment for mother’s smoking and drinking alcohol during pregnancy, parity and social class by including the variables described below into the ordinal linear least-squares regression models, retaining sex and (for models of brain structure outcomes) intracranial volume in the model. Results in Table S1 demonstrate that the associations were stable for additional adjustment.

*Measurements*. Maternal smoking was assessed at the last menstrual period, early pregnancy, and late pregnancy by questionnaire and categorized into a dichotomous variable (yes/no). Maternal alcohol use was assessed at early and late pregnancy by questionnaire and categorized into a dichotomous variable (yes/no). Mother’s socioeconomic class was recorded during the follow-up visit and recoded into five categories using the United Kingdom National Statistics socioeconomic classification. Parity was recoded into four categories (0, 1, 2, and 3 or more births). More information on these measures can be found in an earlier report on this cohort:

Godfrey K, Walker-Bone K, Robinson S, Taylor P, Shore S, et al. (2001) Neonatal bone mass: influence of parental birthweight, maternal smoking, body composition, and activity during pregnancy. J Bone Miner Res 16: 1694-1703.

Spearman correlations showed that none of the potentially confounding covariates was significantly associated with brain volume, attention or inhibitory control (all Ps > .17).

**Table S1.** Results of additional adjustment of regression models that showed significant effects of birth weight on the respective outcome variable.

|  | β | p |
| --- | --- | --- |
| Total brain volume | 0.35 | .001 |
| Total gray matter volume | 0.31 | .009 |
| Total white matter volume | 0.32 | .029 |
| LOFC area | 0.43 | .018 |
| MOFC area | 0.37 | .017 |
| rIFG area | 0.33 | .056 |
| MOFC thickness | -0.42 | .059 |
| Caudate volume | 0.41 | .014 |
| Inhibitory control | 0.47 | .043 |

Note. Adjusted for sex, intracranial volume (except for inhibitory control), mother’s smoking and drinking alcohol during pregnancy, parity and social class. LOFC: lateral orbitofrontal cortex; MOFC: medial orbitofrontal cortex; rIFG: Right inferior frontal gyrus;
